# Outcome of decompressive craniectomy in comparison to nonsurgical treatment in patients with malignant MCA infarction

**DOI:** 10.1186/2193-1801-3-115

**Published:** 2014-02-28

**Authors:** Abdolkarim Rahmanian, Babak Seifzadeh, Ali Razmkon, Peyman Petramfar, Juri Kivelev, Ehsan-Ali Alibai, Juha Hernesniemi

**Affiliations:** Department of Neurosurgery, Shiraz University of Medical Sciences, Nemazee Hospital, P.O. Box: 71937–11351, Shiraz, Iran; Shiraz Neuroscience Research Center, Shiraz University of Medical Sciences, Shiraz, Iran; Department of Neurology, Shiraz University of Medical Sciences, Shiraz, Iran; Department of Neurosurgery, Helsinki University Central Hospital, Topeliuksenkatu 5, 00260 Helsinki, Finland

**Keywords:** Cerebral infarction, Decompressive craniectomy, Middle cerebral artery

## Abstract

**Background:**

Malignant cerebral infarction is a well-recognized disease, comprising 10-15% of all cases with cerebral infarction and causing herniation and death in 80% of cases. In this study, we compare the effects of decompressive craniectomy versus conventional medical treatment on mortality rate and functional and neurological outcome in patients with malignant MCA infarction.

**Methods:**

We performed a prospective case–control study on 60 patients younger than 80years of age suffering malignant MCA cerebral infarction. The case group underwent decompressive craniectomy in addition to routine aggressive medical care; while the control group received routine medical treatment. Patient outcome was assessed using Glasgow outcome scale and modified Rankin scale within three months of follow-up. The data were analyzed by SPSS version 16.0 software using Chi Square, One-way ANOVA and Mann–Whitney tests.

**Results:**

There were 27 male and 33 female patients with a mean age of 60.6 years (SD = 12.3). Glasgow outcome scale score averaged 2.93 in the surgical versus 1.53 in the medical group; this difference was significant (*p* = 0.001). Outcome in modified Rankin scale was also significantly lower in the surgical (3.27) versus medical (5.27) group (*p* < 0.001). Surgery could decrease the mortality rate about 47%.

**Conclusion:**

In this study, decompressive craniectomy could decrease mortality rate, and improve neurological and functional outcome, and decrease long-term disability in patients with malignant MCA infarction.

## Introduction

Malignant supratentorial infarction of the brain is a well-recognized entity, comprising 10-15% of all cases with cerebral infarction. It causes severe edema, increased focal and generalized intracranial pressure, and finally results in transtentorial or uncal herniation in approximately 80% of individuals (Holtkamp et al. 2001; Staykov & Gupta 2011; van der Worp & Kappelle 2011). The optimal management of these patients has been always a challenge. Decompressive craniectomy (DC) has been used for many years as an option but not a standard; while many studies have shown its efficacy in decreasing mortality rates from 80% to 30% (Aarabi et al. 2006; Spagnuolo et al. 2004).

Following the widespread performance of DC in many centers worldwide, newer concerns have been raised. There is still controversy on optimal timing of the procedure, the effectiveness of the operation in different age groups, and most importantly, the significant increase in the severely disabled population of patients who have been added to the survival (Chen et al. 2007; Foerch et al. 2004; Jüttler et al. 2011; Jüttler et al. 2007). Such increase leads to significant health burden and economic pressure over families and the whole society. Until these controversies have not been resolved, the application of DC may be a challenge for every individual patient.

In this study, we compared mortality rates and outcome data in two groups of patients subject to DC or aggressive medical therapy, in population of patients from referral center of Southern Iran with cerebral infarction.

## Methods

We performed a prospective case–control study on patients with massive cerebral infarction of the MCA territory who were admitted consecutivelyto a tertiary center affiliated to Shiraz University of Medical Sciences during 2010 and 2011. Institutional review board and research ethics committee of the Shiraz University of Medical Sciences evaluated and approved the work.

### Patient selection

All consecutive patients with acute presentation of neurologic symptoms, 80 years old or younger, who showed unilateral infarctions of >50% MCA territory in brain CT or MRI during the mentioned period of time were included in the study. Indications for surgery included significant midline shift (>5 mm), effacement of basal cisterns and sulci, and the absence of brain herniation. Absent brain stem reflexes was also a contraindication to enrollment. All patients had to be admitted before 48 hours of presentation. Patients with bilateral infarctions, hemorrhagic infarctions or transformations, and any terminal illness or concomitant severe medical disease contraindicating surgery were excluded. Early coma was not a contraindication to enrollment.

A control group included also consecutive patients who were admitted shortly before the routine application of DC in our center. These patients, fulfilled the above-mentioned criteria of study enrollment, but did not undergo surgery. They had received aggressive medical treatment, and were compared with patients from the case group.

### Treatment

All patients in the control group received aggressive standard medical treatment of cerebral infarction. This treatment included admission to the neurocritical care units, optimization of blood pressure, hyperosmolar therapy, and mechanical ventilation and sedation if necessary. In case of deterioration, no surgical management had been considered for the patients.

All patients in the case group received aggressive medical treatment, as outlined above, in the first step. Surgery was planned after “significant deterioration” and not prophylactically. “Significant deterioration” was defined as further decrease in consciousness to somnolence, stupor or early coma, or progressive development of either unilateral or bilateral pupil abnormalities. As stated earlier, patients with fixed neurological deficits and deep long-standing coma were excluded from the study.

The operation consisted of a large fronto-temporo-parietal craniectomy associated with subtemporal skull decompression. We used a supine position using a roll under the ipsilateral shoulder to avoid over rotation of the head and jugular venous compression. The head was positioned about 10 cm above heart level. We rotated a large traumatic fronto-temporo-parietal skin flap. Five to 6 burr holes were made to design a single bone flap measuring an average of 15 cm in larger diameter. We ensured adequate subtemporal skull decompression to prevent uncal herniation, and remained about 2 cm lateral to the superior sagittal sinus. We opened the dura making a single big dural flap, with a minimum of 5 mm away from bone edges. Duraplasty with pericranium or temporalis fascia was performed. Free bone flap was saved in abdominal subcutaneous space. A subcutaneous drain was inserted and kept until 24 hours after surgery. Aggressive medical management was continued post-operatively in neurocritical care unit. ICP monitoring was not routinely used after surgery. A post-operative CT was performed to rule-out hematoma formation, and ensure adequate decompression. Patients were discharged after becoming neurologically stable, occurring at least one week after surgery.

### Patient evaluation and follow-up

Clinical status was evaluated using GCS scores at presentation. Demographic variables, pre-existing medical conditions and details of neurological examination and imaging were all recorded for further analysis. In-hospital complications, especially those related to the surgical procedure, were all recorded. All patients were followed by both the neurologist and neurosurgeon at three months after discharge. When direct patient visit was not possible, we tried to contact the patient or the visitors by phone to record outcome. Outcome data included Glasgow outcome scale (GOS) score and modified Rankin scale (MRS) at three months after discharge. GOS is used routinely in our center; however, for better clarification of the functional abilities of patients especially with GOS scores of 3 and 4, we added the MRS scale, which gives more differentiating data about the functional status of the patients (Quinn et al. 2009).

### Statistical analysis

All data were analyzed using SPSS software version 16.0. Chi-square, one-way ANOVA and Mann–Whitney tests were used to analyze the data.

## Results

### Patient characteristics

From 83 patients with malignant MCA infarction, 60 patients were considered eligible for inclusion into the study; 30 patients in either case or control groups. Mean age was 60.6 ± 12.3 years (mean ± standard deviation) in both groups. Male to female ration was 0.82. Basic pre-operative clinical parameters have been presented in Table 1. Statistical analysis shows that there was no significant difference between the two groups; therefore, the groups seem matched and comparable.

**Table 1:** **A summary of demographic**, **clinical and radiological parameters in study groups**

Study group	Case (surgical)	Control (medical)	Total	***p*** value
**Number of patients**	30	30	60	
**Mean age ±** **SD** **(years)**	59.0 ± 13.5	62.1 ± 11.0	60.6 ± 12.3	0.44
**Sex** **(male-** **female)**	11 – 19	16 – 14	27 - 33	0.15
**Mean GCSs on admission**	6.9	6.0	6.4	0.29
**Laterality** **(right –** **left)**	18 – 12	21 - 9	39 - 21	0.65

GCS score on admission was assessed using both parametric and non-parametric tests. Mean GCSs was 6.4 (range 4–12); and there was no significant difference between two groups (*p* = 0.29).

When assessing lateralization, 39 patients (65%) had infarctions at the right MCA territory; while 21 (35%) patients developed infarctions at the left side. There was again no significant difference between case and control groups (*p* = 0.65).

In the case group, 18 patients (60%) underwent surgery within 24 hours after presentation; while the remaining 40% underwent surgery in the first 48 hours. Therefore, there was no delayed surgery.

### Outcome data

Table 2 shows a summary of outcome data in both groups. Six patients (20%) died in the DC group, as compared to 20 patients (67%) who died in the control group. Such a difference proved to be significant (*p* < 0.001).

**Table 2:** **Analysis of outcome data in the study groups**

Study group	Case (surgical)	Control (medical)	Total	***p*** value
Number of patients	30	30	60	
Outcome in GCS scale	2.7 ± 1.2	1.5 ± 0.9	2.1 ± 1.2	0.001
Outcome in MRS scale	3.3 ± 1.9	5.3 ± 1.3	4.3 ± 1.9	<0.001
Mortality	20%	67%	43%	<0.001
Complications	-	-	-	0.34

**Figure 1:**
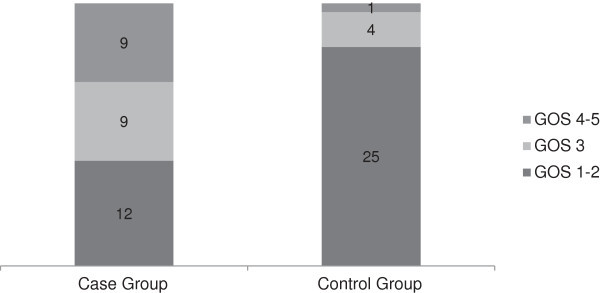
**Outcome at 3 months follow**-**up based on GOS scale.**

GOS scores at three months follow-up were significantly higher (mean: 2.9 vs. 1.5 in case vs. control groups; respectively; *p* = 0.001). When using non-parametric Mann–Whitney test, comparison of ranks proved also significantly different (*p* = 0.001). Figure 1 shows the distribution of different scores in each group.

**Figure 2:**
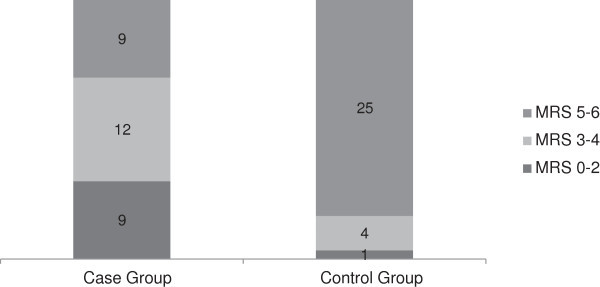
**Outcome at three months follow-** **up based on MRS scale.**

MRS scale was also available for all patients, and similarly, a significant difference was noted. Mean MRS was 3.3 in the case and 5.3 in the control groups (*p* < 0.001). Nonparametric analysis also showed such a significant result (*p* < 0.001). Figure 2 shows the distribution of different scores in each group.

The most common complication encountered during hospital stay was deep venous thrombosis, which occurred in 8.3% of patients, with no significant difference in any of the groups. While all patients received low dose heparin or enoxaparine as a prophylaxis, these two patients underwent vena caval filter placement. Two patients also developed wound infectionsand underwent irrigation and debridement of the wound and received IV antibiotics.

Further analysis could not show any significant difference in outcome when assessing age, sex, laterality and complication rate.

## Discussion

Decompressive surgery has not been previously considered an option in management of patients with malignant MCA infarction. After the introduction of this treatment and its beneficial effects on the prognosis of patients with malignant brain swelling after severe head injury, this treatment has been also introduced for ischemic cerebral strokes (Aarabi et al. 2006; Spagnuolo et al. 2004). The rate of performance of DC has increased tremendously in CVA patients following reports on the beneficial effects of this treatment (Raffelsieper et al. 2002; Swiat et al. 2010; Walcott et al. 2011).

The surgical management of patients with malignant MCA infarction remains a challenge. Even after decades, there is no definitive agreement between experts about the question of whether decompressive surgery should be performed in patients with malignant MCA infarction (Schwab et al. 1998). Decompressive hemicraniectomy can relieve the mass effect due to infarcted brain tissue and prevent brain herniation and death. Several studies have shown that decompressive surgery can reduce the mortality rate from 80 to 30% (Delgado-López et al. 2004; Kakuk et al. 2002; Mellado et al. 2005; Mori et al. 2001; Raffelsieper et al. 2002; Robertson et al. 2004; Swiat et al. 2010; Ziai et al. 2003). There is a significant number of studies in literature with controversial results (Arac et al. 2009; Chen et al. 2007; Foerch et al. 2004; Jüttler et al. 2011; Jüttler et al. 2007). Therefore in this study, we compared the surgical versus non-surgical management of patients with malignant MCA infarctions. There are some important technical points which warrant discussion. The size of the bone flap (Aarabi et al. 2006) must be large enough to allow adequate decompression and prevent pressure at the edges. We considered an average size of 15 cm for the larger diameter of the flap, and visualized no incarceration of the brain tissue at the edges. We also designed a large duraplasty using a single large flap, extending to an average of 5 mm from bone edges. Subtemporal skull decompression was also performed adequately to prevent uncal herniation.

The final outcome measure in our study was mortality showing significant reduction (47%) in the surgically-treated group. The mortality rate was 20% in DC group versus 67% in medically-treated group. This is comparable to many other studies which have uniformly showed a reduction in mortality. Early decompressive hemicraniectomy in patients with malignant MCA infarction reduced the mortality rate by 50% at 1 year compared with best medical treatment in one study (Molina & Selim 2011). Early decompressive craniectomy has decreased mortality to 10-12% in some reports as compared with nonsurgical management with over 50% mortality (Kiphuth et al. 2010; Mellado et al. 2005; Staykov & Gupta 2011; Yang et al. 2005).Our results support previous findings that survival may be better after surgery.

The main challenge is whether the survived patients can return to normal functional life. Some reports have questioned the benefits of decompressive surgery on long-term survival and functional outcome, especially in older patients. Quality of life after hemicraniectomy is one of the most important factors in the choice of treatment for malignant MCA infarction (Cho et al. 2003; Kiphuth et al. 2010; Walz et al. 2002). Some neurosurgeons do not prefer to treat malignant MCA infarction surgically because of concerns about the post-operative quality of life (Weil et al. 2011). There is a lot of controversy in this issue. While some studies have shown that nearly half of patients had good outcome after surgery with GOS scores of 4 and 5 (Mattos et al. 2010; Mori et al. 2004), some others have reported that surgery could not achieve significant good results (Staykov & Gupta 2011; van der Worp & Kappelle 2011). This will increase the number of severely disabled patients, imposing significant pressure over the family and the whole society (Hofmeijer et al. 2009; Schneck & Origitano 2006).

Analysis of our data showed that near 30% of patients in the surgical group had good outcome, i.e. GOS scores of 4 and 5; however, respective rate was only 3.3% in the nonsurgical group. Also, 40% of patients in the DC group and 83% in the control group developed poor outcome (GOS scores of 1 and 2). This means that DC not only decreased mortality but also improved outcome.

Review of literature shows that early surgery (during 24 hours from onset of stroke) has had very good result in all ages especially in younger patients (Koh et al. 2000; Pillai et al. 2007); while surgery later than 48 hours may not benefit patients (Antuña-Ramos et al. 2009; Cho et al. 2003; Chen et al. 2007; Hernández-Medrano et al. 2012; Hofmeijer et al. 2009; Khatri et al. 2008; Schwab et al. 1998; Vahedi et al. 2007; Zhao et al. 2012). We also operated our patients at an early stage (earlier than 48 hours), and therefore, the observed efficacy of DC in our series is perhaps due to this early intervention.

Age is also another influencing factor. Some studies have shown that patients older than 60 had MRS scores of 4 and greater; while patients younger than 60 showed MRS scores of 3 or lower following decompressive hemicraniectomy (Chen et al. 2007; Foerch et al. 2004; Vahedi et al. 2007; Yang et al. 2005). In this study, we did not include patients older than 80, and also could not find any differences between the age groups.

The only problem remains in patients with GOS scores of 3 (MRS of 3 and 4), namely severely disabled patients, which were more frequent in the surgical group (30% versus 13%; respectively). This means that although surgery improved the whole outcome significantly (an average of 1 scores on the GOS and 2 scores on MRS scales) and decreased mortality, survived patients are still in high risk to be severely disabled. Whether this survival is of any benefit to the patients’ families or the whole society is a controversy (Carandang & Krieger 2008; Holtkamp et al. 2001; Kiphuth et al. 2010; Weil et al. 2011). This problem may be considered as a cost-effectiveness issue by health policy makers, or may be presented to the family of any individual patient for decision making. One family may consider even a 1% chance of survival very valuable, while the other one may not accept the continuous expensive care of a severely disabled patient with nearly any chance of improvement. Therefore, consideration of surgical treatment for every patient requires careful consultation with the family members (Hofmeijer et al. 2009; Schneck & Origitano 2006).

A major limitation of the study is the lack of randomization and control of samples. The previously-published beneficial data on effectiveness of DC on mortality (Delgado-López et al. 2004; Kakuk et al. 2002; Mellado et al. 2005; Mori et al. 2001; Raffelsieper et al. 2002; Robertson et al. 2004; Swiat et al. 2010; Ziai et al. 2003) have made the performance of randomized trials impossible. Our data may provide at most level 2 evidence on the benefits of DC; while longer follow-ups may yield more beneficial data.

## Conclusion

Early DC, performed within 48 hours after presentation and on patients younger than 80, could reduce mortality and improve outcome significantly in unilateral malignant MCA infarction. We recommend performing the procedure for every eligible patient after careful consultation with the family members.

## Consent

Written informed consent was obtained from all patients for the publication of this report and any accompanying images.
